# Editorial: The pharmacotherapy of depression—searching for new mechanisms and drug interactions basic and clinical research

**DOI:** 10.3389/fphar.2022.1098660

**Published:** 2022-11-30

**Authors:** Katarzyna Stachowicz, Magdalena Sowa-Kućma, Anna Tabecka-Łonczyńska

**Affiliations:** ^1^ Department of Neurobiology, Maj Institute of Pharmacology, Polish Academy of Sciences, Kraków, Poland; ^2^ Department of Human Physiology, Institute of Medical Sciences, Medical College of Rzeszow University, Rzeszow, Poland; ^3^ Department of Biotechnology and Cell Biology, Medical College, University of Information Technology and Management in Rzeszow, Rzeszow, Poland

**Keywords:** depression, pharmacotherapy, new mechanisms, new drugs interactions, side effects

Recognizing the importance of depression in this particular volume of Frontiers in Pharmacology, we invited the authors to highlight new concepts in the pharmacotherapy of depression. According to the WHO, the COVID-19 pandemic has caused a massive increase in the global prevalence of anxiety and depression. Pharmacotherapy of depression is mainly based on substances first discovered and synthesized in the 1960s and 1970s; hence there is a great need to define new molecular pharmacological targets and interactions between administered drugs to increase treatment efficacy and reduce side effects characteristic of currently available antidepressants. This problem was described by Stachowicz and Sowa-Kućma. In addition, a structured state of knowledge of what is happening at the preclinical and clinical levels is presented in a two-part review by (Vasiliu). The first part of this review focuses on monoaminergic, orexinergic, GABA-ergic, and anti-inflammatory agents with antidepressant activity. Many of the antidepressants described are currently being marketed. In addition, new drugs are being developed, and psychoactive substances and drugs previously introduced to the pharmaceutical market are also being used. Orexin receptor modulators and unused medications from the group modulating the properties of GABA-A receptors/neurosteroid analogs are showing promising results. Anti-cytokine therapies and COX-2 inhibitors also demonstrate antidepressant properties, and the benefits of biological treatments should not be overlooked (Vasiliu). The second part of Vasiliu’s review deals with clinical trials of antidepressants, among which are non-monoaminergic substances—esketamine for treatment-resistant major depression and brexandone for postpartum depression. The best-known group are glutamatergic agents: antagonists of NMDA receptors or their GluN2B subunits, AMPA receptor enhancers, and metabotropic receptor ligands. Combinations of pharmacological agents are also being studied. On this basis, it can be concluded that the development of new therapies offers high hopes for the effective treatment of depression shortly (Vasiliu). Another review paper also provides no less exciting data by Zhao et al., which details the current state of research on the molecular, cellular, and neuronal mechanisms of dopamine (DA) receptors involved in depression, including the types of receptors and their differential distribution in the brain. Importantly, research progress on the role of D1-D2 heterodimers in depression is also presented (Zhao et al.).

High hopes for finding new, better, and more effective antidepressant therapies are offered primarily by the original studies presented in this issue, and they often explore completely new areas. For example, Meng et al. demonstrated the antidepressant properties of LPM570065 (a potent triple 5-HT/NE/DA reuptake inhibitor) in mice that experienced “two-hit” stress and described the mechanisms involved in this effect. In addition to an increase in the density of dendritic spines in hippocampal CA1 neurons after LPM570065 administration, interesting epigenetic mechanisms were described. Two-hit stress-induced changes in the mouse hippocampus, such as hypermethylation and downregulation of the OXTR (oxytocin receptor) gene along with increased levels of DNA methyltransferases proteins (Dnmt1 and Dnmt3a), were reversed by LPM570065 (Meng et al.). In addition, Lebeau et al. detected electroconvulsive seizure-responsive proteins in a rodent model unresponsive to chronic fluoxetine. These included cell adhesion, cytoskeletal, coagulation, and those involved in regulating immune responses. Similarly, studies in a rat model of chronic unpredictable mild stress (CUMS) using honokiol as the biologically active substance extracted from *Magnolia Officinalis* demonstrated its antidepressant effects (Fan et al.). These effects were attributed to VEGFR2-mediated activation of HIF-1α-VEGF and PI13K/AKT/mTOR signaling and increased expression of proteins associated with synaptic plasticity: SYN1 and PSD95. These results were also confirmed by *in vitro* studies in which honokiol increases synaptic plasticity in PC12 cells through activation of the HIF-1α-VEGF signaling pathway (Fan et al.).

Amylin receptors (AMYRs) are a novel target under investigation for depression (Jiang et al.). AMYRs are dimers of calcitonin receptors (CTRs) with receptor activity modifying proteins (RAMPs). Their potential in depression has been verified using agonists (salmon calcitonin) and antagonists (AC187) of AMYRs in mice.

Herbs and folk medicine preparations (in this case, Chinese medicine) interest researchers. In this case, Yang et al. described the antidepressant activity of an ancient Chinese formula called Xiaoyaosan. Its antidepressant activity, among many, involves the GLUT4 pathway and autophagy mechanisms.

Monitoring side effects and drug-drug interactions is essential in the search for new antidepressants. Such a significant problem of antidepressant-drug interactions with over-the-counter (OTC) drugs is described by Woroń et al. More than four percent of antidepressant side effects were associated with OTC, particularly omeprazole, diphenhydramine, ginkgo Biloba, ibuprofen, and diclofenac. In addition, Miziak et al. discussed the co-occurrence of epilepsy and depression and how to successively treat both diseases, excluding side effects and drug interactions.

As can be seen, the problem under discussion was treated very comprehensively, and thanks to the efforts of the authors, reviewers, and editors, it was possible to offer a passionate and novel compendium on depression.

